# The mechanics of male courtship display behaviour in the *Ptiloris* riflebirds (Aves: Paradisaeidae)

**DOI:** 10.1093/biolinnean/blae077

**Published:** 2024-09-16

**Authors:** Thomas MacGillavry, Clifford B. Frith, Leonida Fusani

**Affiliations:** 1 Konrad Lorenz Institute of Ethology, University of Veterinary Medicine, Vienna, Austria; 2 PO Box 581, Malanda, QLD 4885, Australia; 3 Department of Behavioural and Cognitive Biology, University of Vienna, Vienna, Austria

**Keywords:** display behaviour, courtship, birds of paradise, sexual selection

## Abstract

Sexual selection through female choice has driven the evolution of some of the most elaborate signalling behaviours in animals. These displays often require specialized morphological adaptations and may incorporate signals in multiple sensory modalities. Visual and acoustic signals are often precisely choreographed in temporally structured courtship performances, though the precise mechanics of such signalling behaviours are often enigmatic. We find that riflebirds (genus *Ptiloris*)—a bird of paradise clade—achieve their remarkable display postures by hyperextending the wrist joint, vastly exceeding the maximal wrist extension capabilities of any other known bird. Using video collected in the field, we then show that this hypermobility is required for a sonation unique to riflebirds, and find that the yellow interior of the mouth is displayed in the dynamic phase of display. As this sonation cannot be produced when the mouth is exposed, it represents a mechanical constraint to signal design. Finally, we used a large morphometric dataset to describe patterns of sexual dimorphism in wing length across diverse bird of paradise species, and find evidence of sexual selection for large and structurally modified wings used in riflebird displays. Our study highlights nuanced choreographic differences in the display behaviours of different riflebird species, and sheds light on the intricate design features of sexual signals in this fascinating taxon.

## Introduction

Birds have evolved a remarkable diversity of sexual signalling behaviours. While courtship displays often consist of simple, repetitive movements, some species perform intricate display choreographies that frequently involve the precise coordination of both acoustic and visual signals (e.g. Dalziell *et al*. 2013, Ota *et al*. 2015). Sustained mate choice selecting for such signalling behaviours may result in the evolution of specialized or exaggerated morphological adaptations that enhance the efficacy of displays (Darwin 1871, Bostwick *et al*. 2012, Friscia *et al*. 2016, Fuxjager *et al*. 2016). Since signalling behaviours can evolve to remarkable extremes and often involve postures and movements that have little or no function in other contexts, sexual selection represents a key driver of phenotypic innovation. To understand the evolution of complex signalling behaviours, it is therefore essential to study their mechanical and morphological bases. While much research has aimed to describe general patterns of courtship behaviours among birds, we know comparatively little about the fine-scale elements of elaborate courtship performances.

While the birds of paradise (Aves: Paradisaeidae) present a textbook example of elaborate courtship performances, few detailed studies have been conducted on their display behaviours. The parotias (genus *Parotia*) represent a noteworthy exception, where systematic attempts at documenting their courtship behaviours have revealed a remarkable choreographic complexity in their displays (Scholes 2006, 2008a, b, c). While recent efforts at documenting the display behaviours of all bird of paradise species using video footage collected in the wild have greatly enhanced our understanding of sexual signalling behaviours in these enigmatic birds (e.g. Ligon *et al*. 2018), detailed knowledge about the display behaviours of most species is scarce.

**Figure 1:**
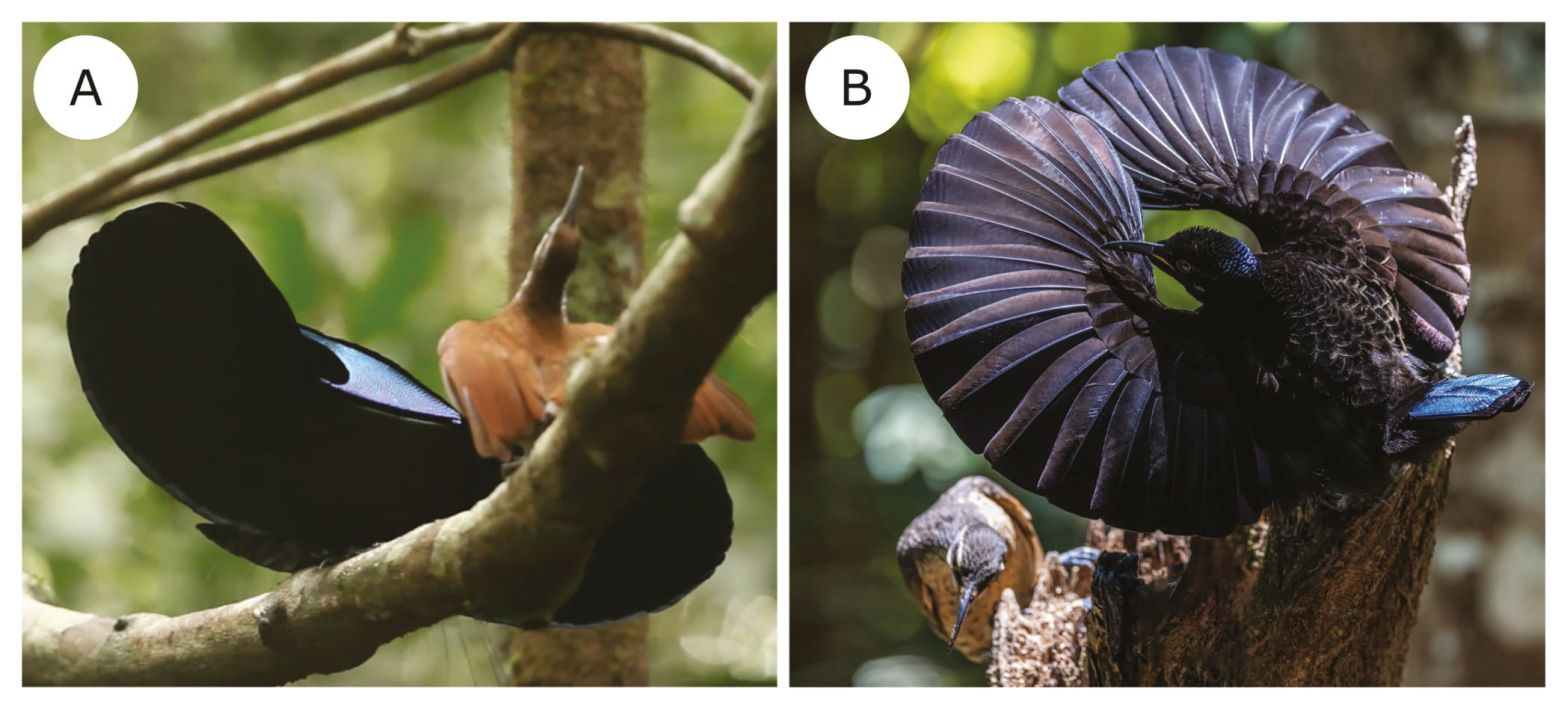
Riflebirds in display showing a spread wings posture in the magnificent riflebird (A) and circular wings display in Victoria’s riflebird (B). Images in A and B were sourced from the Macaulay Library [accession number and image credits: ML455444 (still taken from video), Edwin Scholes; ML384020521, Jill Duncan & Ken Bissett, respectively].

The riflebirds (genus *Ptiloris*), for example, encompass four species found in rainforest habitats in New Guinea and Australia. Victoria’s riflebird *Ptiloris victoriae* and the paradise riflebird *P. paradiseus* are the only two species endemic to Australia, while the growling riflebird *P. intercedens* is endemic to Papua New Guinea. The magnificent riflebird *P. magnificus* is widespread in New Guinea, but can also be found in rainforests in the Cape York Peninsula of Australia (Beehler and Swaby 1991). All riflebird species are polygynous, exhibiting extreme sexual plumage dimorphism and delayed plumage maturation in males, which are strikingly black with blue or blue-green iridescent coloured feather patches (Frith and Beehler 1998). In contrast, females and immature males of all species are varying shades of brown, and females are not known to perform elaborate sexual displays. Males of all riflebird species perform unusual wing postures and rhythmic, alternating wing and head motions during courtship (Frith and Cooper 1996, Frith and Beehler 1998; Fig. 1). In Victoria’s riflebird, these alternations are associated with striking clap-like sonations, which are thought to be produced as the primary feathers overlap and presumably collide between alternations, hence why this phase of courtship has been termed the ‘wing-clap’ display by Frith and Cooper (1996). Sonations such as these are widespread in birds, and have been well researched in the manakins (Pipridae)—which snap their wings together behind their backs or against their flanks (Bostwick 2000, Bostwick and Prum 2003, Fusani and Schlinger 2012, Bodony *et al*. 2016)—and hummingbirds (Trochilidae)—which utilize the aeroelastic properties of their tail and wing feathers to create chirping sounds during courtship flights (Clark 2008, 2011, *et al*. 2013). In the riflebirds, however, little is known about the sonation mechanisms incorporated in display, and the courtship performances of this clade have never been investigated systematically.

The purpose of the present study is twofold. We first aimed to determine the precise mechanical features of display behaviour in Victoria’s riflebird and congeneric species using video footage and morphological measurements collected in the field. Second, we used a large, international museum specimen morphometrics dataset to investigate whether sexual selection drove the evolution of sexual dimorphism in wing length in this clade, where the wings exhibit a key signalling function. To this end, we investigated both general patterns of sexual dimorphism across the bird of paradise radiation, as well as finer patterns within the riflebird genus. We find that riflebird display behaviours are much more mechanically intricate than previously anticipated, exhibiting several features unique to the genus *Ptiloris*. Furthermore, we find evidence of intense sexual selection upon wing length in this remarkable avian clade.

## Materials and Methods

### Study site and banding activities

We studied the display behaviour of male Victoria’s riflebird in upland rainforest in the Atherton Tablelands in Queensland, Australia (17°15′55″S, 145°,37′47″E). Victoria’s riflebirds were captured with mist-nets placed near display perches and leg-banded with two to three plastic-coloured bands and a single-numbered metal band to identify individuals. Banding was conducted with the approval of the Australian Bird and Bat Banding Scheme (R-class banding authority number: 3662). Ethical approval was granted by the Animal Ethics Committee of the Department of Agriculture and Fisheries (AEC reference number: CA 2022/02/1589). Field activities at private properties and the Crater Lakes National Park (17.283°S, 145.625°E) were approved by the Queensland Wildlife and Parks Service (P-PTUKI-100257238; WA0045747). Bird capture and banding activities in Kutini-Payamu (Iron Range) National Park in the Cape York Peninsula (Queensland, Australia) were performed under an ongoing project (national parks permit number: P-PTUKI-100156548; ethics permit number: CA2020-11-1435). Since permits in Kutini-Payamu (Iron Range) national park did not cover biological sampling, individuals trapped in this locality could not be genetically sexed. Permits for field work in the Atherton tablelands only permitted blood sampling of Victoria’s riflebirds, which was not possible for the collected individual as it was found dead (see below).

### Wing measurements and riflebird wrist mobility in a phylogenetic context

We opportunistically collected a specimen of a female-plumaged individual Victoria’s riflebird found dead in the field. We then measured the maximum angle of the manus by placing markers on the terminal end of the manus, ulnar–metacarpal (‘wrist’) joint, and ulnar–humeral (‘elbow’) joint, and measured the angle of the wrist using ImageJ (Rueden *et al*. 2017). Maximum wrist extension was measured by extending the manus until resistance was met, being careful not to damage the joints (see Baliga *et al*. 2019).

We further measured one living adult female (sexed by the presence of a brood patch) and one living unsexed, female-plumaged magnificent riflebird in Kutini-Payamu (Iron Range) National Park in the Cape York Peninsula by photographing the hyperextended wing with fingers indicating joint positions (see Supporting Information). One bird was only photographed from the dorsal side and the second only from the ventral side as we found this allowed us to inspect the positions of the joints more clearly. Despite this inconsistency, measurements of both birds were highly similar (see Supporting Information Table S1). Furthermore, while we did not genetically sex the female magnificent riflebird it is highly unlikely that this individual was an immature male, as male incubation behaviour, especially by an immature male, has never been documented in any polygynous bird of paradise species. To account for measurement uncertainty, we measured each photograph three times and report the mean of these three measurements in subsequent text and figures.

Measurements were taken following a simplified version of the protocol described in Baliga *et al*. (2019); we measured range of motion by mounting a wing to a Styrofoam block and actuating manus extension by hand, which we then photographed from above. To measure the wrist angle, we marked the tip of the manus, wrist, and elbow joints with adhesive markers. We only made exception with the live-caught individuals, as placing markers on the wing joints would substantially increase handling time and additionally stress the animals. Instead, we held the birds in a way that allowed us to indicate the position of the relevant joints by hand, while an assistant photographed the bird. While Baliga *et al*. (2019) quantified multiple axes of joint mobility of multiple joints in three dimensions—including abduction and rotation of the manus—we were only interested in quantifying the degree of wrist extension in two dimensions. Since we only measured this single axis of mobility in one joint, we consider these measurements comparable to those previously published.

We also tested for the presence of hypermobility in one live-caught, unsexed trumpet manucode *Phonygammus keraudrenii*—a sexually monomorphic and socially monogamous bird of paradise (Frith and Beehler 1998). However, due to time constraints in the field, we could not take photographs in which the exact extension of the wrist could be measured (see Supporting Information Fig. S5).

**Figure 2:**
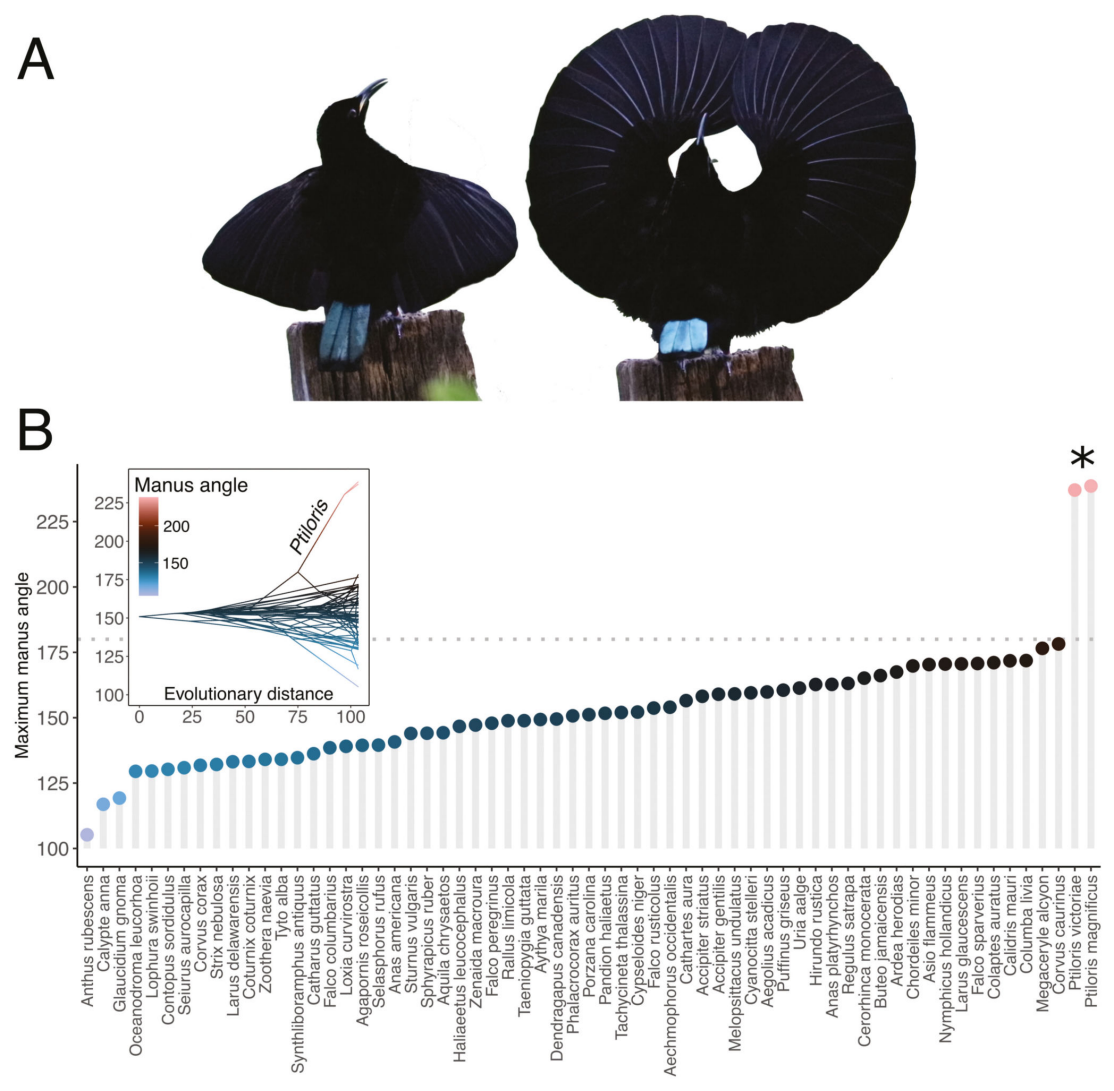
Victoria’s riflebird performs an unusual display posture by raising the wings and hyperextending the wrist joint (A). Compared to other bird species (*N* = 61), Victoria’s riflebird and the magnificent riflebird are the only species capable of wrist hyperextension, where the wrist is extended more than 180° (B). The asterisk indicates more than five SD degrees of extension above control species means (*P. victoriae* = 5.42 SD, *P. magnificus* = 5.52 SD). The horizontal dotted line indicates 180° maximal wrist extension and units on the *y*-axes are in degrees. Images in A were taken from video footage collected by T.M.

To compare the maximal wrist angles of riflebirds to those of other bird species, we sourced a previously published wing mobility dataset (Baliga *et al*. 2019). To produce the phenogram shown in Figure 2B, we downloaded 2000 subtrees from the Ericson supertree from Jetz *et al*. (2012). We then created a 50% majority rule consensus tree in Geneious Prime 2021.1.1 (https://www.geneious.com/). This tree contained one polytomy, which was resolved randomly using the ‘*fix.poly*’ function in the R package *RRphylo* (Castiglione *et al*. 2018). Ancestral maximal manus angle states were estimated using the ‘*fastAnc*’ function it the R package *phytools* (Revell 2024). All statistical analyses were performed in R v.4.4.0 (R Core Team 2020).

### Video footage of displaying riflebirds

To investigate general features of display in Victoria’s riflebird, we installed motion-triggered trail cameras (Browning Recon Force Advantage HD; Browning Recon Force Elite HP5) at display perches. Overall, we inspected 1191 min of camera trap video footage showing adult males displaying to receivers (MacGillavry *et al*. 2024), considerably extending the amount of video used in previous descriptions (Frith and Cooper 1996). We also opportunistically collected video footage of three displaying adult male Victoria’s riflebirds (60 fps with audio and 120 fps without audio) using a DSLR (Canon 5D mark 4 fitted with a 100–400-mm lens) placed at a known distance from the display perches. We further obtained an additional high-speed video (240 fps) filmed using an iPhone 12 pro mounted to a scope (Swarovski ATX85) from J.d.R. (see Acknowledgements). We further corroborated previously published descriptions of displaying magnificent and paradise riflebirds using video footage available in the Macaulay Library (Cornell Lab of Ornithology: https://www.macaulaylibrary.org/).

### Sexual selection on wing length across the birds of paradise

To investigate patterns of sexual dimorphism across the birds of paradise, we used a large morphometric dataset encompassing specimens from a global sample of museum collections (Frith and Frith 1997). To explore variation in relative male wing length (measured as the chord length of the folded wing) and sexual dimorphism in relative wing length across the birds of paradise (*N* = 39 species), we first estimated ancestral states as described above. Relative wing length was calculated separately for each sex by taking the residuals from a phylogenetic log_10_–log_10_ regression of wing length against tarsus length by implementing phylogenetic generalized least squares (PGLS; correlation= ‘‘corBrownian’’) using the ‘*gls*’ function in the R packages *nlme* (Pinheiro *et al*. 2023) and *ape* (Paradis and Schliep 2019) using the bird of paradise tree topology available in Ligon *et al*. (2018). This topology was modified from Irestedt *et al*. (2009) by placing the superb lophorina *Lophorina superba* as a sister to *Ptiloris*, based on more recent phylogenetic analyses (Irestedt *et al*. 2017). Since this modified tree was no longer ultrametric, we ultrametricized the topology by extending branches with the ‘*force.ultrametric’* function in the R package *phytools* (Revell 2024). We then calculated sexual dimorphism in residual wing length by subtracting female values from male values (Clark and Rankin 2020). For information on how tarsus length and wing length were measured, see Frith and Frith (1997).

Tarsus length generally represents a useful proxy of body size in birds (Killpack and Karasov 2012). Since body mass data were not available for most specimens, and may not be comparable for museum specimens due to differences in specimen preparation and species differences in elaborate plumage characters, we consider tarsus length to be the most accurate metric of body size available in our dataset. While tarsus length has been found to correlate relatively weakly with body size compared to other skeletal measurements, it is probably a reasonably accurate proxy for body size among closely related taxa that exhibit similar flight behaviours (Field *et al*. 2013), which is the case for the birds of paradise.

We then tested whether riflebirds exhibited signs of sexual selection for wing length using PGLS (correlation = “corPagel”) and ordinary least squares (OLS) models. For PGLS models, Pagel’s lambda was estimated using maximum likelihood. We hypothesized that species in the genus *Ptiloris* should exhibit (i) larger wings relative to body size in males and (ii) more sexual dimorphism in relative wing length compared to other birds of paradise. Sexual dimorphism in relative wing length describes differences between the sexes within species and therefore better reflects variation that may result from sexual selection rather than the potential influence of natural selection on differences in relative wing length between species. To test this, we fitted models with either residual male wing length or sexual dimorphism in residual wing length as response variables (see above) and taxon—including the monogamous clade (consisting of the manucodes [genera *Phonygammus* and *Manucodia*] and paradise crow [genus *Lycocorax*]), core bird of paradise species not including riflebirds, and the riflebirds (genus *Ptiloris*, which is nested within the core Paradisaeidae)—as a predictor.

### Relative wing length and sexual dimorphism in riflebirds

We further used the measurements from Frith and Frith (1997) to investigate the intraspecific allometric scaling relationships for males and females in the four riflebird species (magnificent riflebird *P. magnificus*, growling riflebird *P. intercedens*, Victoria’s riflebird *P. victoriae*, and paradise riflebird *P. paradiseus*) using multiple regression. We first fit a full model using wing length as the response variable and tarsus length, sex, and species as predictors, including a three-way interaction between each variable, as we expected that (i) wing length should increase with body size, (ii) sexual dimorphism in wing length resulted in different scaling relationships with body size between the sexes, and (iii) these differences varied between species. To ease the interpretability of model coefficients, we *z*-transformed tarsus length to a mean of zero and a standard deviation of one using the ‘*scale*’ function in R. In the magnificent riflebird and Victoria’s riflebird, plots suggested that each had one influential outlier among females, which we identified and removed by means of *z*-scores using the ‘*check_outliers*’ function in the R package *performance* (Lüdecke *et al*. 2021).

We inspected a qq-plot of the residuals and the residuals plotted against fitted values. We then assessed collinearity among predictors using generalized variance inflation factors (Fox and Monette 1992) computed using the ‘*vif*’ function in the package *car* (Fox *et al*. 2023). Our model including all riflebird species showed clear signs of collinearity among predictors. To avoid issues due to multicollinearity, we chose to run separate models for each of the four species. None of these individual models showed signs of multicollinearity or obvious deviations from the assumptions of normally distributed and homogeneous residuals. Finally, we assessed model stability using DF-beta (Nieuwenhuis *et al*. 2012) computed using functions available in R (see ‘max’ and ‘min’ values in Tables 1 and 2).

For each species, we performed full–null model comparisons by comparing the fit of a full model with that of a null model including all terms except in which the interaction term between sex and tarsus length was omitted. This approach is preferred over assessing the fit of all possible combinations of predictors, which results in ‘cryptic multiple testing’ (Forstmeier and Schielzeth 2011). Overall, our final sample included 114 magnificent riflebirds (*N*_males_ = 68, *N*_females_ = 46), 47 growling riflebirds (*N*_males_ = 23, *N*_females_ = 24), 80 Victoria’s riflebirds (*N*_males_ = 50, *N*_females_ = 30), and 58 paradise riflebirds (*N*_males_ = 34, *N*_females_ = 24). We only included adult specimens where both tarsus length and wing length measurements were available, and where sex was known (Frith and Frith 1997).

## Results

### Riflebird wrist mobility

In the birds we measured, the angle of the manus at maximal extension measured at 237.1° in Victoria’s riflebird (unsexed) and 238.9° in the magnificent riflebird (female *P. magnificus* = 236.6°, unsexed *P. magnificus* = 240.6°; see Supporting Information Table S1). This vastly exceeds the maximal wrist extension abilities of any other known bird species, none of which in our dataset were capable of hyperextension (i.e. extension beyond 180°). Victoria’s riflebird and the magnificent riflebird exhibit maximal manus angles of 5.42 and 5.52 standard deviations above the mean maximal manus extension of the control species, respectively.

While the exact maximal wrist angle could not be measured in the trumpet manucode, it is likely that this species cannot massively hyperextend the wrist (Supporting Information Fig. S5), though it appears that an angle of ~180° could be achieved, which is comparable to the northwestern crow *Corvus caurinus*, which also exhibits a relatively high degree of wrist mobility compared to other species in the dataset (Fig. 2B). However, since this individual was unsexed, we lack conclusive evidence to suggest that male trumpet manucodes do not exhibit wrist hypermobility.

### Sonations and novel choreographic features of Victoria’s riflebird display

**Figure 3:**
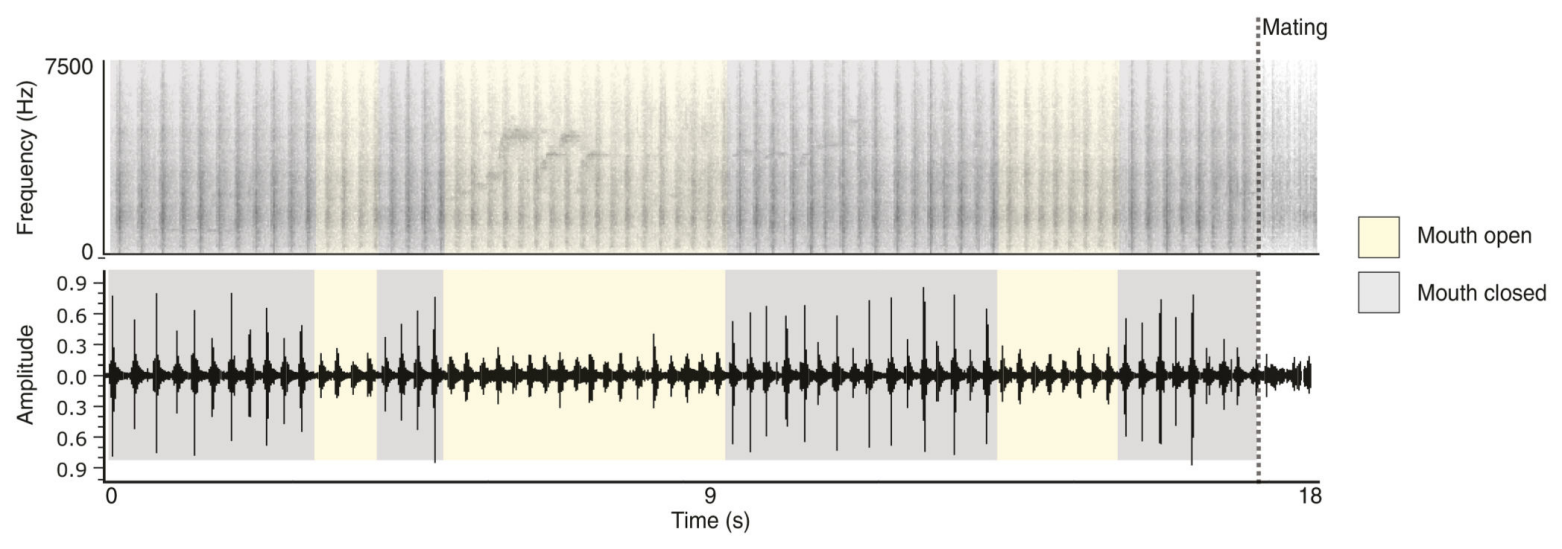
Spectrogram of the high-intensity phase of a wing-clap display in Victoria’s riflebird illustrating the loss of the ‘snap’ sonation when the mouth is opened. The wings continue to produce a conspicuous rustling sound when the mouth is open.

**Figure 4:**
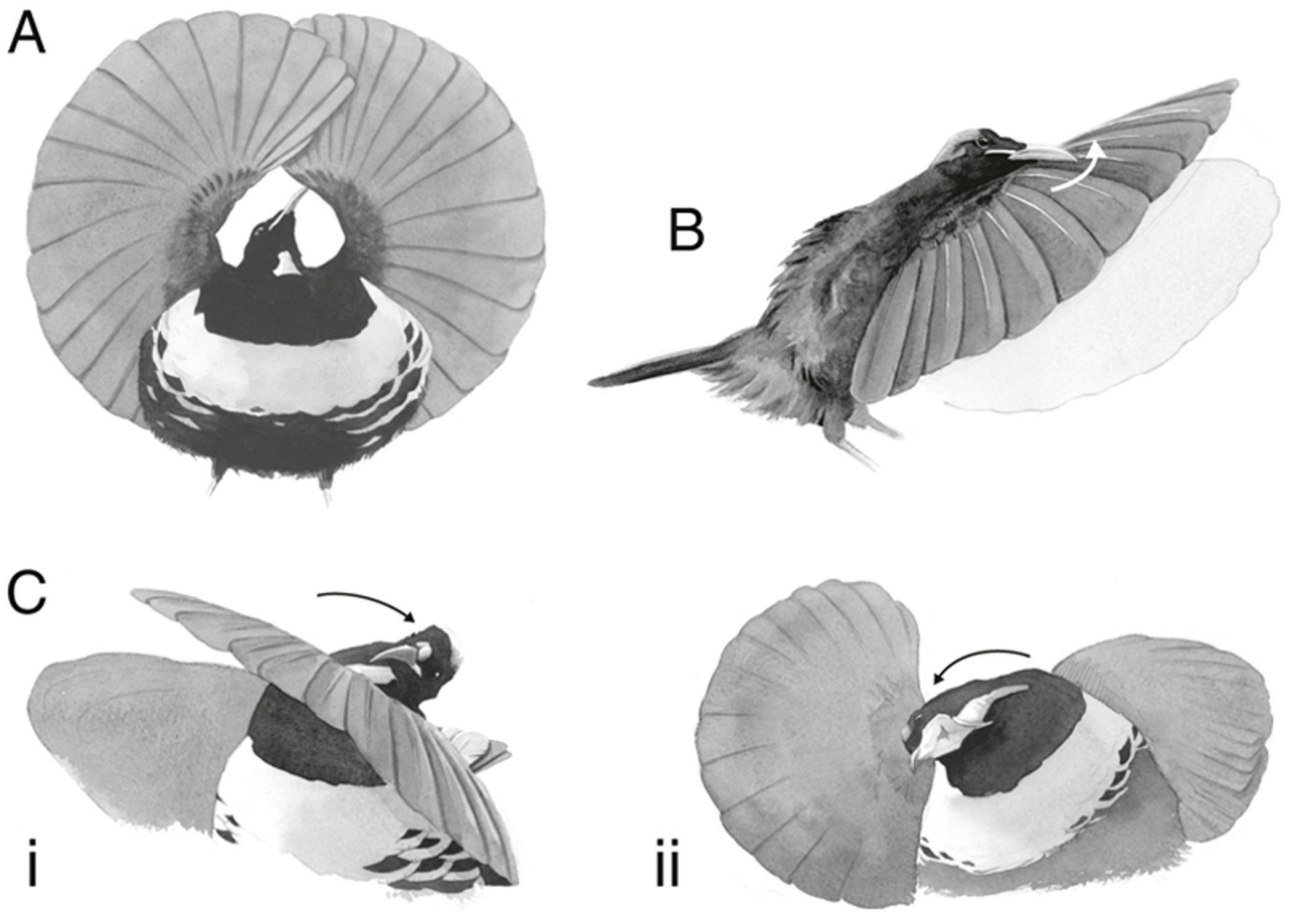
Summary of novel display features in the courtship display of Victoria’s riflebird described here. Male Victoria’s riflebirds perform a striking *circular-wings* display, involving extreme wrist hyperextension (A). During the dynamic phase of display, they produce a snap-like sonation by scraping the bill upward across the primary feathers (the white arrow indicates the trajectory of the bill as it scrapes along the dorsal surface of the wing; B). During the ‘wing-clap’ display, the head and wings are alternated in opposing motions (C.i). When displaying at high intensity, the bill is opened, exposing the yellow gape, though this creates a mechanical conflict with the bill-scraping sonation (C.ii). Watercolour paintings were kindly provided by Joris de Raedt.

Our high-speed video footage (Supporting Information Video S1) shows that, contrary to previous descriptions (Frith and Cooper 1996, Frith and Beehler 1998), Victoria’s riflebird sonates by scraping the bill across the dorsal surface of the wing (see Figs 3 and 4 for details). While all riflebirds produce conspicuous rustling noises in the dynamic phase of display (Frith and Beehler 1998, see Macaulay Library accession number: ML481867; https://www.macaulaylibrary.org/), we find that Victoria’s riflebird additionally incorporates a *bill-scraping* sonation (Fig. 4; see Video S3).

Using our camera trap dataset, we further found that, during the dynamic phase of display, the bill is occasionally opened to display the yellow interior of the gape (see Supporting Information Video S4). The presentation of the gape was previously recognized to be an important component of the static *circular wings* phase of display (Frith and Cooper 1996), though it was not known to also occur during the dynamic *alternating wing-clap* phase, possibly due to the quality of video footage previously available.

Given the mechanism of sonation described above, Victoria’s riflebirds cannot simultaneously display the gape and perform a high-amplitude sonation by bill-scraping. While we lack detailed acoustic data of this complex display, we visualized this effect in Figure 4, which clearly shows that, when the gape is exposed, the amplitude of each ‘clap’—coinciding with a single alternation—is greatly reduced. Wing-rustling, though less conspicuous, persists as a low-amplitude sonation when the bill is opened. Conspicuous feather rustling such as this has been described in other bird of paradise species during flight (Coates 1990), though riflebirds appear to be one of few species where it may also have a signalling function during display.

### Interspecific patterns of sexual dimorphism

Our ancestral character reconstructions indicate that, while male riflebirds do not exhibit notably increased relative wing lengths compared to other bird of paradise species (Fig. 6A), the magnificent and growling riflebirds exhibit the most extreme sexual dimorphism in relative wing length (Fig. 6B). Interestingly, some species (e.g., the parotias) exhibited both relatively short wings in males as well as a female-biased sexual dimorphism in relative wing length, suggesting that mate choice may select for wing size in diverse ways depending on their signalling function.

Our quantitative analysis of sexual dimorphism in wing length provided a more detailed perspective. While riflebirds did not exhibit larger relative male wing lengths than the monogamous clade (OLS; estimate ± SE = −0.0011 ± 0.0297, *P* = .970; see Table 1), the relative wing length of males in other core bird of paradise species was smaller than in the monogamous clade(OLS; estimate ± SE = −0.0724 ± 0.0206, *P* = .001). However, this effect was not statistically significant when accounting for phylogenetic relatedness (PGLS; estimate ± SE = −0.0782 ± 0.0485, *P* = .115, λ_ML_ = 0.926). Furthermore, we found that, while the core bird of paradise species in general did not show more or less sexual dimorphism than the monogamous clade (OLS; estimate ± SE = 0.0058 ± 0.0060, *P* = .343), the riflebird genus exhibited significantly more sexual dimorphism in relative wing length than the monogamous clade (OLS; estimate ± SE = 0.0339 ± 0.0087, *P* < .001). However, this effect was also not statistically significant when accounting for phylogenetic relatedness (PGLS; estimate ± SE = 0.0299 ± 0.0156, *P* = .063, λ_ML_ = 0.735).

### Intraspecific patterns of sexual dimorphism

We found no evidence of an interaction between tarsus length and sex in the magnificent riflebird (full-null model comparison: *F*_1,110_ = 0.339, *P* = .562; Fig. 6A) and growling riflebird (full-null model comparison: *F*_1,43_ = 0.967, *P* = .331; Fig. 6B). However, in the Victoria’s riflebird (full-null model comparison: *F*_1,76_ = 6.716, *P* = .011; Fig. 6C) and paradise riflebird (full-null model comparison: *F*_1,54_ = 8.96, *P* = .004; Fig. 6D), we found evidence that the scaling relationships of wing length versus tarsus length differed between the sexes, as the interaction between sex and tarsus length significantly improved the model fit.

Interestingly, wing size scaled less sharply with body size for males compared to females in Victoria’s riflebird (estimate for tarsus length for males ± SE = −2.242 ± 0.865, *P* = .011; Fig. 6C) and the paradise riflebird (estimate for tarsus length for males ± SE = −2.888 ± 0.965, *P* = .004; Fig. 6D). In the growling riflebird, wing length did not appear to scale with tarsus length in either sex (estimate for tarsus length ± SE = −0.387 ± 1.331, *P* = .772; Fig. 6B; Table 2).

## Discussion

Sexual selection through female mate choice is a salient evolutionary process that has generated a remarkable diversity of signalling behaviours among animals. In few organisms are such displays as elaborate as in the birds of paradise. It is therefore unsurprising that this enigmatic avian clade has inspired naturalists and scientists alike since well before Darwin’s time (Darwin 1871, Gilliard 1969, Frith and Beehler 1998). Despite a long history of scientific interest, we currently lack a detailed understanding of the fine-scale components of male courtship displays in most bird of paradise species, which inhabit remote rainforest habitats in New Guinea, the Northern Molukkas, and Australia (Frith and Beehler 1998). In the riflebirds, the general patterns of display behaviour have been well described in the literature (Gilliard 1969, Frith and Cooper 1996, Frith and Beehler 1998). Our study has nonetheless revealed that these performances are much more mechanically intricate than previously anticipated, and highlights how mate choice can drive the evolution of extreme behavioural and morphological phenotypes that function exclusively for sexual display.

### The mechanical basis of display in riflebirds

Male riflebirds perform elaborate, rhythmic courtship displays that involve the intricate coordination of diverse motor components (Frith and Cooper 1996, Frith and Beehler 1998). We found that these display behaviours rely on the ability to massively hyperextend the wrist joint. When compared to a previously published dataset spanning diverse neognathes (Baliga *et al*. 2019), riflebird wrist mobility vastly exceeds the joint range of motion of any other known bird species with the exception of one artificially selected pigeon breed—the Ukrainian skycutter *Columba livia* (Zilberg and Cryberg 2008) (Fig. 2B). Since skycutter pigeons were previously found to hyperextend the wrist up to ~30°, compared to nearly 60° in both riflebird species measured here, riflebirds exhibit the most extreme wrist-joint mobility of any known bird species.

While we lack wing mobility data for a broader set of bird of paradise species, it is likely that the trumpet manucode—a socially monogamous species—is not capable of extreme wrist hyperextension (Supporting Information Fig. S5). This suggests that this ability evolved in the core bird of paradise radiation (Irestedt *et al*. 2009) and may indeed be unique to riflebirds. Since the manucode we observed could not be sexed, we cannot, however, definitively conclude that males cannot hyperextend the manus in this species. Future work should therefore aim to measure additional, sexed specimens, encompassing both male and female individuals. While we were also not able to measure wrist extension in adult male riflebirds, wing extension in our Victoria’s riflebird specimen qualitatively closely resembled that of displaying adult males (see Fig. 2a; Fig. S2). Our measurements therefore probably closely reflect the capabilities of adult males.

Within the riflebirds, we also note nuanced interspecific differences in which wrist hyperextension is incorporated in display (Table 3). Wrist hypermobility is not required for the static phase of display in the magnificent riflebird (see Fig. 1A), but does visibly occur during the dynamic phase. In Victoria’s riflebird (and the paradise riflebird, Macaulay Library accession number: ML465654), wrist hyperextension is required for sonation during the *wing-clap display*, whereby the wrist of one wing is hyperextended at a time, and rhythmic movements are performed by alternating which wing is extended coincident with alternations of the head (and thus presenting the iridescent blue throat patch; e.g. Macaulay Library accession number: ML456288; Supporting Information Videos S1–S4). In the magnificent riflebird, however, only the head is alternated and the wrists are briefly hyperextended as they are flicked upwards, creating a rustling sound (e.g. Macaulay Library accession number: ML455444). There are therefore nuanced differences in the ways male Victoria’s and magnificent riflebirds incorporate wrist hyperextension into their displays (Table 3).

Surprisingly, we found that one live-caught adult female magnificent riflebird also exhibited extreme wrist mobility (Fig. 7). Similarly, females of certain manakin species in which males sonate through wing-snapping—such as the club-winged manakin *Machaeropterus deliciosus*—possess similarly (though to a lesser extent) modified wing bones as males (Bostwick *et al*. 2012, Bodony *et al*. 2016). One possible explanation for the expression of specific male morphological traits associated with sexual display in females is genetic correlations between the sexes (Clark and Rankin 2020). Since female magnificent riflebirds are not known to perform the characteristic courtship displays of this species, their ability to hyperextend the wrist may represent a case of pleiotropy. The sexually dimorphic expression of courtship phenotypes may largely be regulated by sex differences in hormone production and the tissue-specific expression of androgen receptors (Fusani 2008, McQueen *et al*. 2021, Fuxjager *et al*. 2022), though different traits may be more or less under precise endocrine control. While difficult to test in nonmodel organisms, sexual dimorphism in skeletal or joint characteristics may stem from less precise sex-biased endocrine control compared to plumage or behavioural traits. Nonetheless, we found wrist hyperextension to interact with additional features of male display.

In Victoria’s riflebird, we found that wrist hyperextension is required for a unique mode of sonation: the bill is scraped across the rachises of the flight feathers along the dorsal surface of the wing (specifically the stout bases of the primary feather rakises; see Supporting Information Video S1), creating a conspicuous ‘snap’ sound. Considering the perspective of female riflebirds, due to the close proximity of display, it is possible that sonations produced by successive alternations during display differentially stimulate each ear, though this warrants further study. Such acoustic features further appear to interact with additional visual elements, such as the presentation of the inside of the mouth or ‘gape’.

We find that, when the gape is presented during the dynamic phase of display, the bill is first moved to the inner margin of the wing before the mouth is opened (Fig. 4c; Supporting Information Video S2). In this position, the head is tilted towards the receiver and the bill can no longer be used to stridulate, thus creating a situation where either the bill is scraped across the wing to produce a sonation, or the gape is displayed. While it appears that the ‘snap’ sound is not produced consistently, even when the bill is closed, it is completely absent when the gape is displayed as a result of this mechanical conflict (Fig. 3). More sophisticated methods are required to determine whether this apparent inconsistency in sonation represents a real biological phenomenon, or whether the direction of displaying males greatly influences the amplitude of the signal when recorded from a fixed point (but not from the perspective of an attending female). Nonetheless, the mechanism of sonation in Victoria’s riflebird—which relies on wrist hyperextension—creates a mechanical trade off with the *gape-flash* display, thus constraining signal design in this species (see Table 3 for a comparison with other riflebird species). In addition to the subtle choreographic differences in display behaviour, we found evidence of selection on wing length in male riflebirds.

### Sexual dimorphism in wing length in riflebirds and other birds of paradise

**Figure 5:**
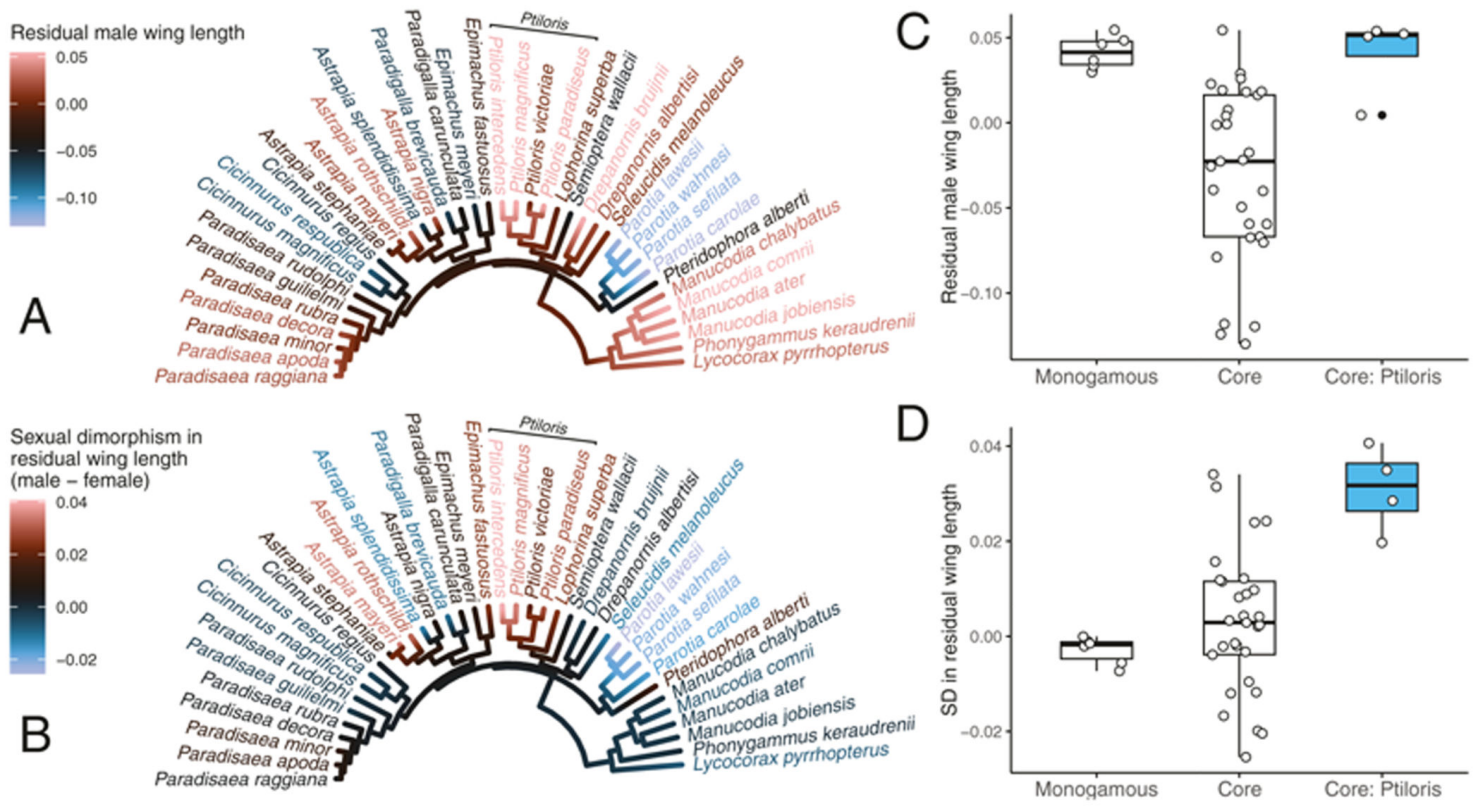
Ancestral character estimation of residual male wing length (A) and sexual dimorphism in residual wing length (B) plotted on a bird of paradise phylogeny. Sexual dimorphism in residual wing length was greatest in *P. magnificus* and *P. intercedens*. We further investigated differences in residual male wing length (C) and sexual dimorphism (‘SD’) in residual wing length (D) using PGLS and OLS models (see Table 1). Box plots show the median (black line), interquartile range (box), minimum and maximum value within 1.5 times the interquartile range of the box (whiskers), and outliers (filled circles). The blue boxes highlight these statistics for the riflebird genus and white circles represent raw data points.

**Figure 6:**
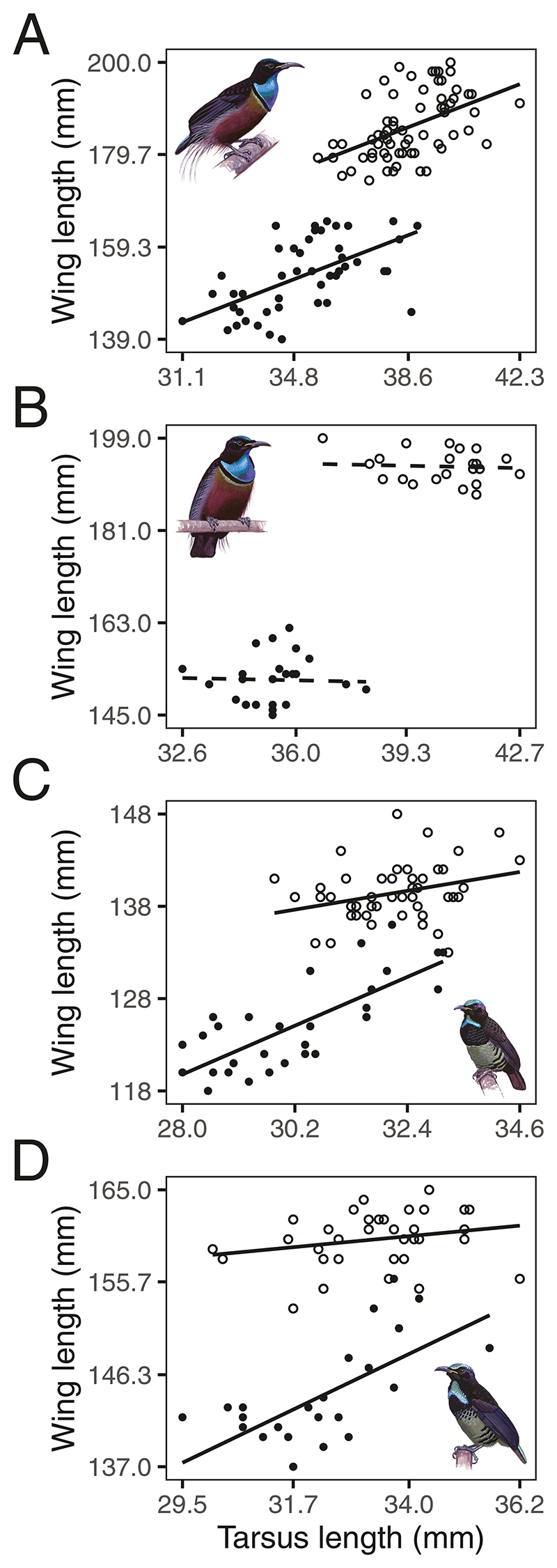
The relationship between tarsus length and wing length between males and females shows evidence of extreme sexual selection. Empty circles show male values and filled circles show female values. In the Magnificent riflebird (A) and growling riflebird (B), we found no evidence that wing length scaled differently with body size between the sexes, while we did find support for this interaction in Victoria’s riflebird (C) and the paradise riflebird (D). Interestingly, we found no significant correlation between wing length and tarsus length in the growling riflebird (indicated by the dashed regression line). Regression lines are based on fitted models (see Table 2).

**Figure 7:**
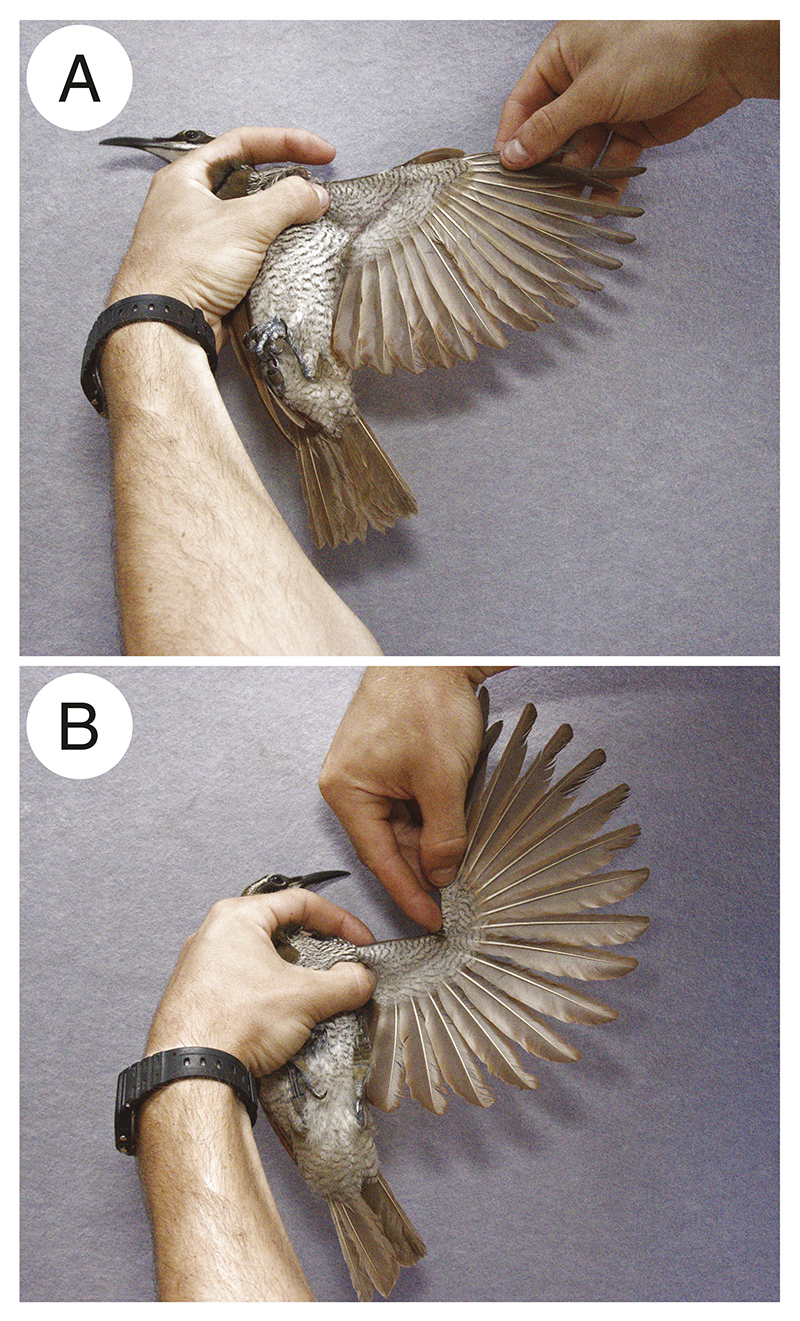
Wrist hypermobility in an adult female magnificent riflebird *P. magnificus* (sexed by the presence of a brood patch). A, the wing extended normally, approximating its position during gliding flight; B, the maximal extension ability of the wrist.

Interestingly, male riflebirds do not appear to have evolved notably large wings relative to their body size when compared to the socially monogamous manucodes and paradise crow, and the ‘core’ birds of paradise exhibit smaller relative wing lengths compared to both riflebirds and monogamous paradisaeids. However, this effect is lost when accounting for phylogenetic relatedness (Fig. 5A, B; Table 1). As variation in relative wing length between species may be shaped by numerous forms of social or natural selection, we cannot conclude a clear role of sexual selection on wing size based on these measurements. However, we also found tentative evidence that riflebirds exhibit extremely male-biased sexual dimorphism in relative wing length compared to other bird of paradise species (Fig. 5C, D), which suggests that sexual selection through female mate choice has had a marked effect on wing size in male riflebirds. However, while the riflebird genus as a whole exhibits some of the greatest sexual dimorphism in relative wing length among the birds of paradise—with the greatest values in the family exhibited by the magnificent riflebird and growling riflebird—the results of our comparative analyses do not conclusively demonstrate that the genus as a whole has experienced extreme selection on male wing length, as this effect is lost when accounting for phylogenetic relatedness.

This may be explained by the nuanced differences in display behaviour between Victoria’s riflebird and the paradise riflebird—which display at extremely close distances to attending females—and the magnificent riflebird (and probably also the growling riflebird)—where the distance between the displaying male and attending female appears to be much greater (see Supporting Information Video S4 and Macaulay Library accession number: ML455444). If the perceptual function of an enhanced wing length is to cover a greater portion of the female’s field of view, then selection on increased wing length will be stronger when the distance between signaller and receiver increases. However, this remains speculative, and intraspecific patterns of sexual dimorphism support the hypothesis that wing length and, by extension, wing surface area play an important signalling role in riflebirds.

**Figure 8:**
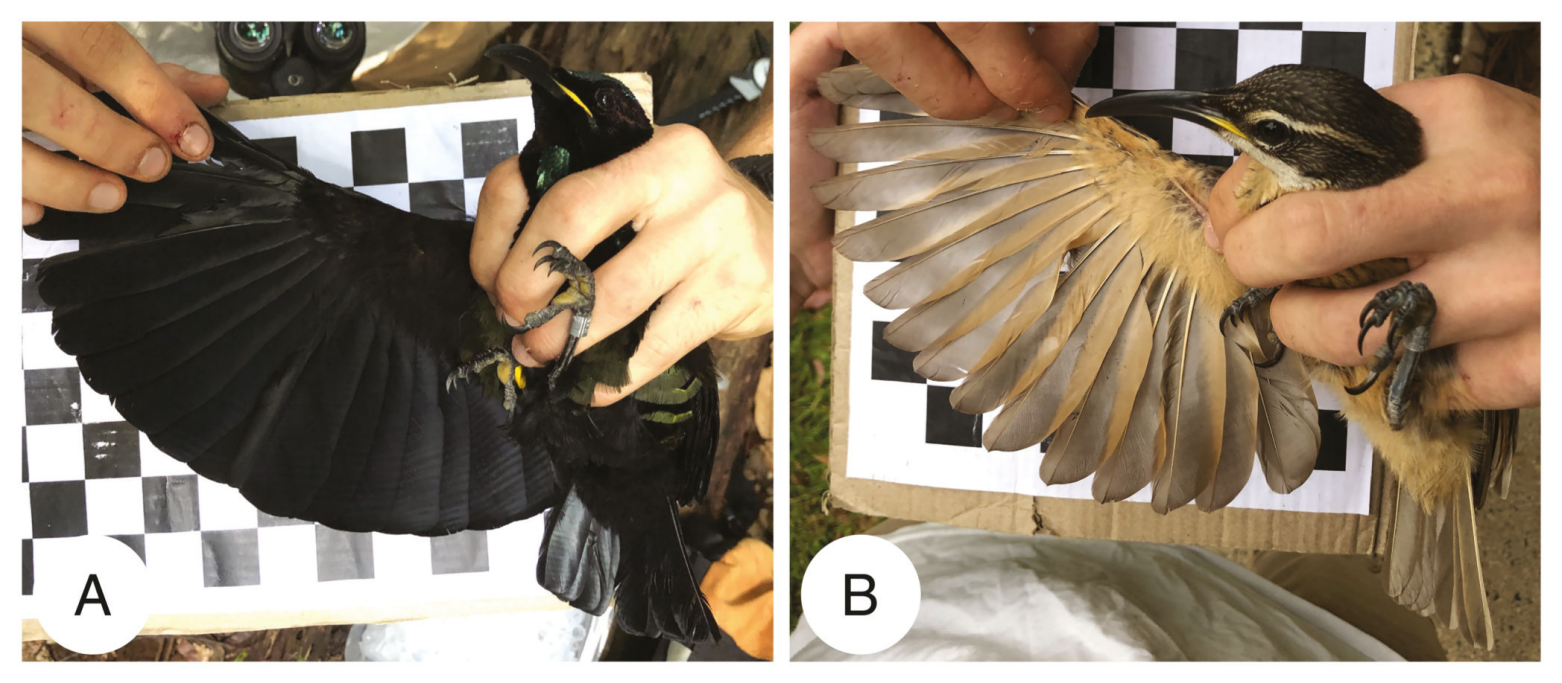
Photographs of adult male (A) and immature male (B) Victoria’s riflebirds show major differences in the structure of the flight feathers. Compared to immature males, the primary feathers of adult males are conspicuously square-ended, which probably serves a signalling function. We included a checkerboard (2.4 mm) as a rough reference for scale.

In each riflebird species, allometric scaling relationships of wing length with body size for males are shifted relative to females. In each species, with the exception of the growling riflebird, wing length scaled with body size, though males exhibited much greater wing lengths than females. The case of the growling riflebird is intriguing, as a lack of a correlation between wing length and body size may suggest that (i) wing size and body size scaling are decoupled or (ii) tarsus length does not accurately represent body size in this species. Either possibility is intriguing, as tarsus length did correlate with wing length in all other riflebird species, including the closely related magnificent riflebird. Moreover, wing length scaled less steeply with body size in males compared to females in both Victoria’s riflebird and the paradise riflebird. This may be indicative of a constraint to wing length in these species. While we lack the data to test this, complex interactions between wing size, body size, and flight mechanics may differ between the sexes, as males possess highly modified flight feathers that may further affect flight mechanics. It is further unclear, however, why we did not find this to be the case for the other riflebird species. Future research on the relationship between flight mechanics and both wing size as well as the unusual, modified flight feathers of adult male riflebirds (Fig. 8; Jackson 1920) will clarify the potentially conflicting effects of both sexual selection and natural selection on riflebird morphology. Furthermore, sexual dimorphism may result from differential niche partitioning between the sexes (Selander 1966), though this explanation is unlikely as there is little indication that male and females riflebirds occupy different foraging strata (Grant and Litchfield 2003) or exhibit clear differences in flight behaviour. However, the putative sexual dimorphism in bill length among riflebirds suggests that males and females may indeed occupy different foraging niches, though this has as yet not been demonstrated and thus remains speculative (Frith 1997). Overall, our finding that males exhibited much larger wings independently of their body size across all riflebird species points to a role of sexual selection in the evolution of the wings of male riflebirds.

## Conclusions

Our study has revealed a striking mechanical complexity in the courtship display behaviours of male riflebirds. Video footage and morphological measurements collected in the field led to the unexpected finding that display behaviour in the riflebirds relies on the unique ability to hyperextend the wrist joint, which is at present not known to occur in any other bird species. High-speed video of Victoria’s riflebird further clarifies the role of wrist hyperextension in sonation; by hyperextending the wing, the bill can be scraped along its dorsal surface to produce a conspicuous snapping sound. However, this sonation cannot be produced when the bright yellow interior of the mouth is shown in later stages of display, creating a mechanical constraint to multimodal signalling.

As in other polygynous bird of paradise species, the elaborate nature of riflebird display has in all likelihood resulted from a long history of sexual selection through female mate choice (Irestedt *et al*. 2009). We found tentative evidence that mate choice drove the evolution of extremely sexually dimorphic wings in the riflebird genus; while our analyses of interspecific differences in wing sexual dimorphism are inconclusive, intraspecific patterns in wing length allometry indicate that wing size may be an indicator of male quality or enhance the efficacy of sexual signalling, or both (Andersson 1994, Endler and Basolo 1998). An emphasis on quality indicators, however, ignores the perceptual functions of specific design features of riflebird display, which are integrated into temporally structured performances (Frith and Cooper 1996, Frith and Beehler 1998). To understand the design features of elaborate sexual signalling behaviours in riflebirds, future research should be aimed directly at understanding the temporal organization of courtship display behaviour.

## Supplementary Material

Supplementary information

VideoS1

VideoS2

VideoS3

VideoS4

## Figures and Tables

**Table 1:** Outputs of PGLS and OLS models investigating differences in either residual male wing length or sexual dimorphism in residual wing length between different bird of paradise taxa (*N* = 39 species).

Response	Model	Predictor	Estimate	Lower CI	Upper CI	SE	*t*-value^a^	*P*-value^a^
Residual male wing length	PGLS	(Intercept)	0.0389	−0.0306	0.1083	0.0355		
	(λ = 0.926)	Taxon = Core	−0.0782	−0.1731	0.0168	0.0485	−1.6132	.115
		Taxon = Core: Ptiloris	−0.0150	−0.1246	0.0947	0.0559	−0.2679	.790
	OLS	(Intercept)	0.0415	0.0047	0.0783	0.0188		
		**Taxon = Core**	−**0.0724**	−**0.1128**	−**0.0320**	** 0.0206**	−**3.5096**	** .001**
		Taxon = Core: Ptiloris	−0.0011	−0.0593	0.0571	0.0297	−0.0379	.970
Sexual dimorphism in residual wing length	PGLS	(Intercept)	−0.0033	−0.0226	0.0161	0.0099		
	(λ = 0.735)	Taxon = Core	0.0042	−0.0218	0.0302	0.0133	0.3144	.755
		Taxon = Core: Ptiloris	0.0299	−0.0007	0.0605	0.0156	1.9172	.063
	OLS	(Intercept)	−0.0029	−0.0137	0.0078	0.0055		
		Taxon = Core	0.0058	−0.0060	0.0176	0.0060	0.9604	.343
		**Taxon = Core:Ptiloris**	** 0.0339**	** 0.0169**	** 0.0508**	** 0.0087**	** 3.9128**	**<.001**

For PGLS models, λ was estimated using maximum-likelihood (‘ML’, see methods). Parameters with *P*-values that were deemed significant based on a significance threshold of 0.05 are shown in bold, and *t*-values and *P*-values for intercepts are not shown due to limited interpretability_a_.

**Table 2:** Outputs of models investigating scaling patterns between body size and wing length between the sexes.

Species	Predictor^a^	Estimate	Lower CI	Upper CI	SE	*t*-value^b^	*P*-value^b^	Min.	Max.
Magnificent riflebird	(Intercept)	158.416	155.985	160.847	1.227			157.510	158.698
	**Tarsus**	** 5.971**	** 4.212**	** 7.731**	**0.888**	** 6.725**	**<.001**	**5.411**	**6.097**
	**Sex = M**	** 23.774**	** 20.203**	** 27.344**	**1.802**	**13.194**	**<.001**	**23.450**	**25.020**
Growling riflebird	(Intercept)	151.539	148.685	154.393	1.416			150.773	152.166
	Tarsus	−0.387	−3.071	2.296	1.331	−0.291	.772	−1.270	−0.177
	**Sex = M**	** 42.290**	** 36.980**	** 47.600**	**2.635**	**16.051**	**<.001**	**41.472**	**44.134**
Victoria’s riflebird	(Intercept)	128.049	126.644	129.455	0.706			127.792	128.494
	**Tarsus**	** 3.652**	** 2.540**	** 4.764**	**0.558**	** 6.541**	**<.001**	**3.459**	**3.929**
	**Sex = M**	** 10.745**	** 8.989**	** 12.501**	**0.882**	**12.185**	**<.001**	**10.300**	**11.002**
	**Tarsus: Sex (M)**	** −2.242**	** −3.964**	** −0.519**	**0.865**	**−2.591**	** .011**	**−2.548**	**−1.914**
Paradise riflebird	(Intercept)	145.702	144.190	147.214	0.754			145.288	146.269
	**Tarsus**	** 3.601**	** 2.139**	** 5.063**	**0.729**	** 4.938**	**<.001**	**3.087**	**4.028**
	**Sex = M**	** 14.078**	** 12.138**	** 16.018**	**0.968**	**14.547**	**<.001**	**13.511**	**14.492**
	**Tarsus: Sex (M)**	** −2.888**	** −4.822**	** −0.954**	**0.965**	**−2.993**	** .004**	**−3.331**	**−2.374**

Tarsus length was *z*-transformed to a mean of zero and a standard deviation of one^a^. t-values and *P*-values for intercepts are not shown due to limited interpretability^b^. Parameters with *P*-values that were deemed significant based on a significance threshold of 0.05 are shown in bold.

**Table 3:** Summary of current knowledge on the display behaviours of three riflebird species: Victoria’s riflebird *P. victoriae*, paradise riflebird *P. paradiseus*, and magnificent riflebird *P. magnficus*.

Courtship display behaviour	Display phase	Riflebird species		
		*P. victoriae*	*P. paradiseus*	*P. magnificus*
Wrist hyperextension	Static	+	+	−
Wrist hyperextension	Dynamic	+	+	+
Gape presentation	Static	+	+	− (?)
Gape presentation	Dynamic	+	?	− (?)
Head alternation	Dynamic	+	+	+
Wing movement	Dynamic	Alternating	Alternating	Simultaneous
Bill-scraping sonation	Dynamic	+	+ (?)	− (?)
Movement away from/towards female	Dynamic	−	−/+	+
Erect posture with folded wings	Static	−	−	+
Synchronous hopping	Dynamic	−	−	+
Typical display perch	Static + dynamic	Vertical stump	High horizontal branch	Low horizontal branch

Since very little is known about the display behaviour of the growling riflebird *P. intercedens*, we did not include it here. However, since this species used to be considered a subspecies of magnificent riflebird and differs primarily in its vocal behaviour, its display repertoire is probably highly similar to that of the magnificent riflebird.

## Data Availability

The data and R code required to replicate the analyses presented here can be found in GitHub (https://github.com/ThomasMac1998/Riflebird_mechanics/tree/main).
