# Supplementary material for: The mechanics of male courtship display behaviour in the Ptiloris riflebirds (Aves: Paradisaeidae)
Source: Biol J Linn Soc Lond. Author manuscript; Available in PMC 2025 Apr 30. (PMC7617636; doi:10.1093/biolinnean/blae077)
Supplement: Supplementary information [file EMS204722-supplement-Supplementary_information.docx]

*Supplementary information*

**The Mechanics of Male Courtship Display Behaviour in the *Ptiloris* Riflebirds (Aves: Paradisaeidae)**

Thomas MacGillavry^1^, Clifford B. Frith^2^, & Leonida Fusani^1, 3^

^1^Konrad Lorenz Institute of Ethology, University of Veterinary Medicine, Vienna, Austria

^2^P.O. Box 581, Malanda QLD 4885, Australia

^3^Department of Behavioural and Cognitive Biology, University of Vienna, Vienna, Austria


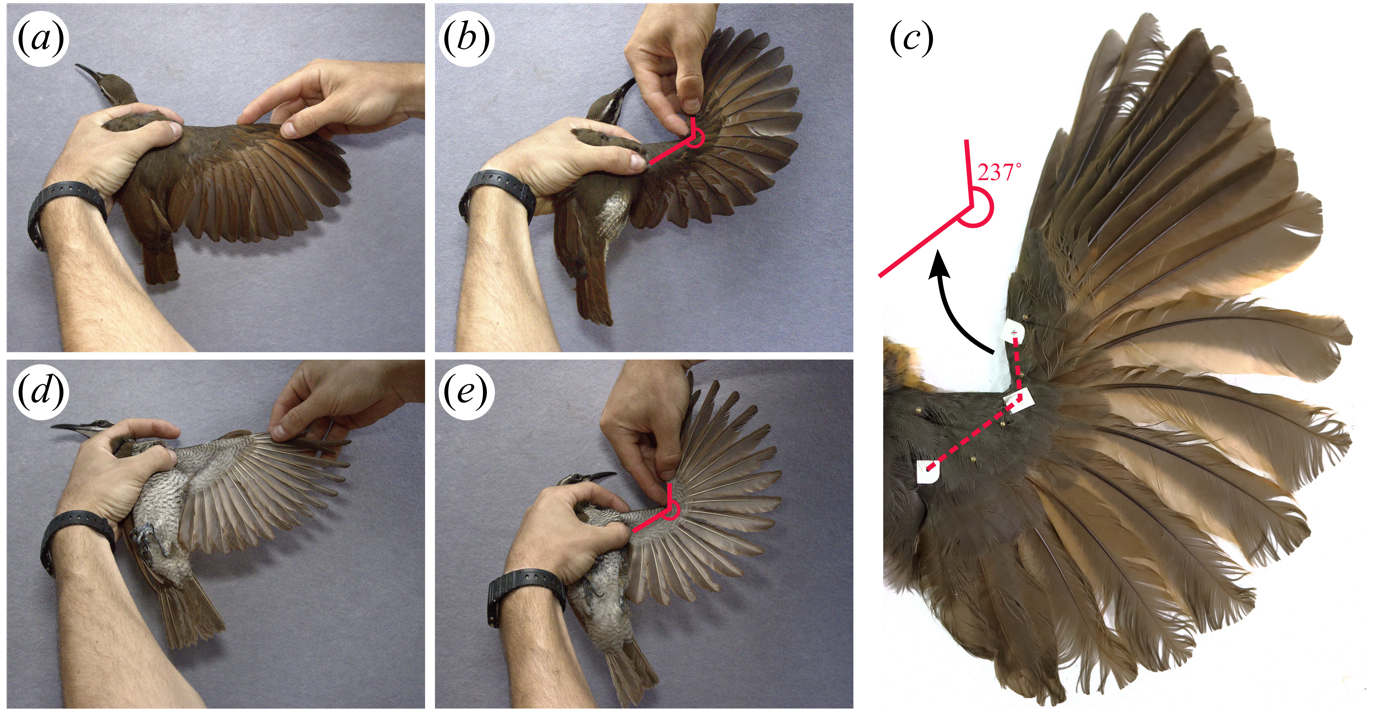


**Figure S1.** Images showing approximately from where wrist extension was measured in three riflebirds. Panels (a) and (b) show a dorsal view of an unsexed (probably immature male) Magnificent Riflebird with wing outstretched normally versus hyper-extended, respectively. Panels (d) and (e) show the same for an adult female magnificent riflebird. Panel (c) shows how wrist hyper-extension was measured in an unsexed female-plumaged Victoria’s Riflebird specimen, with stickers indicating the exact positions of relevant joints (see methods).


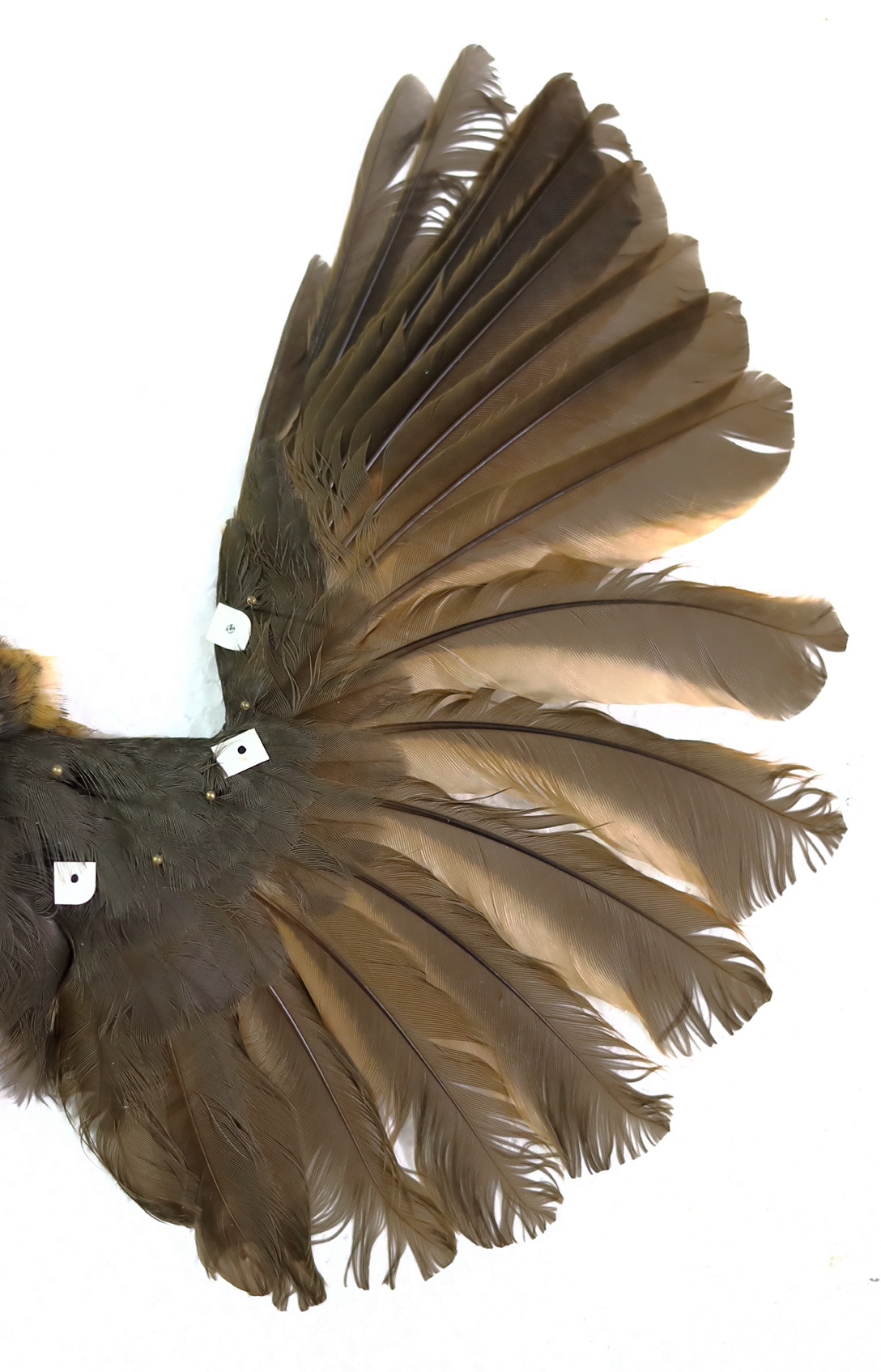


**Figure S2.** Unedited photograph of the Victoria’s Riflebird specimen that was measured.


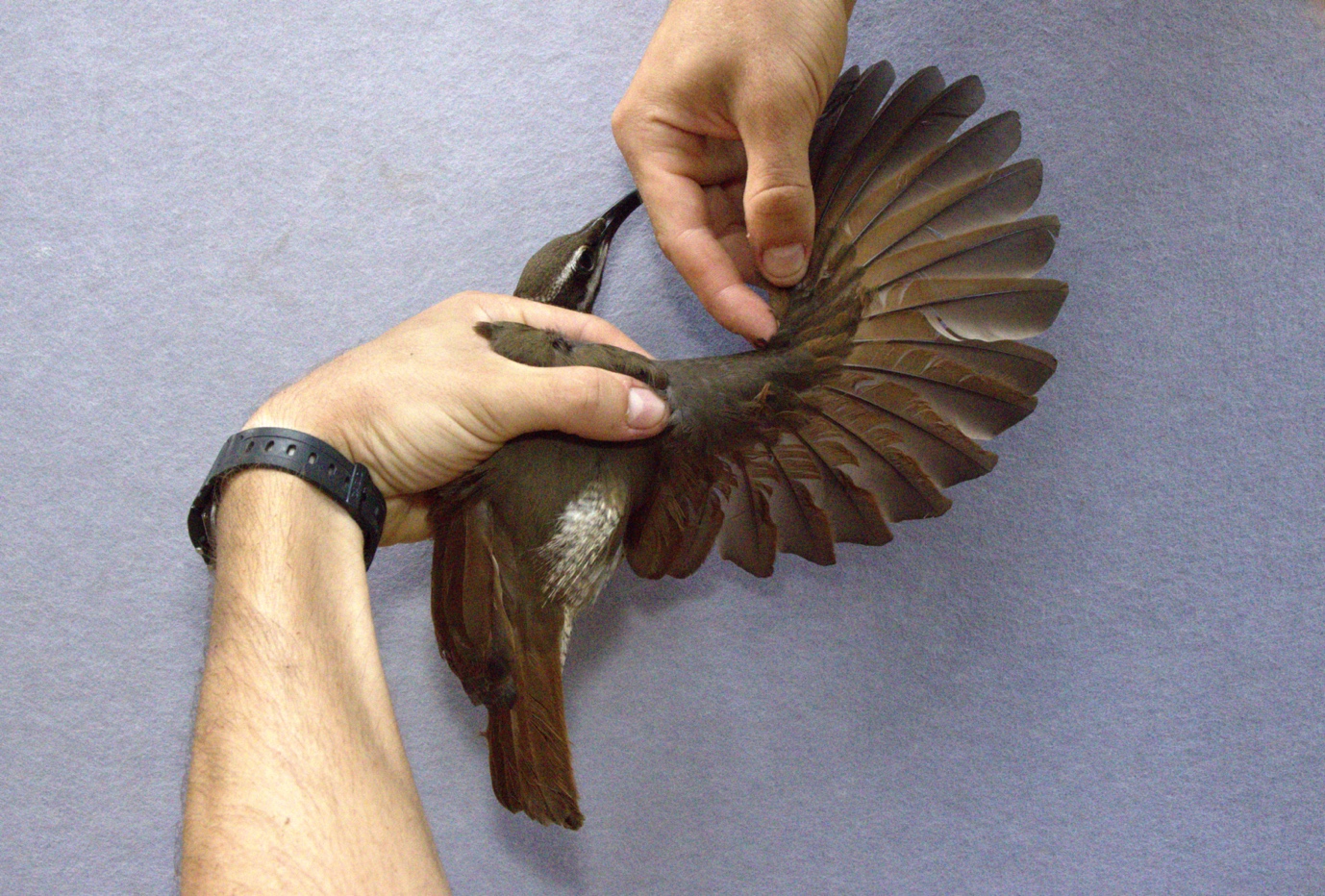


**Figure S3.** Unedited photograph of the unsexed female-plumaged Magnificent Riflebird that was measured with wing hyper-extended.


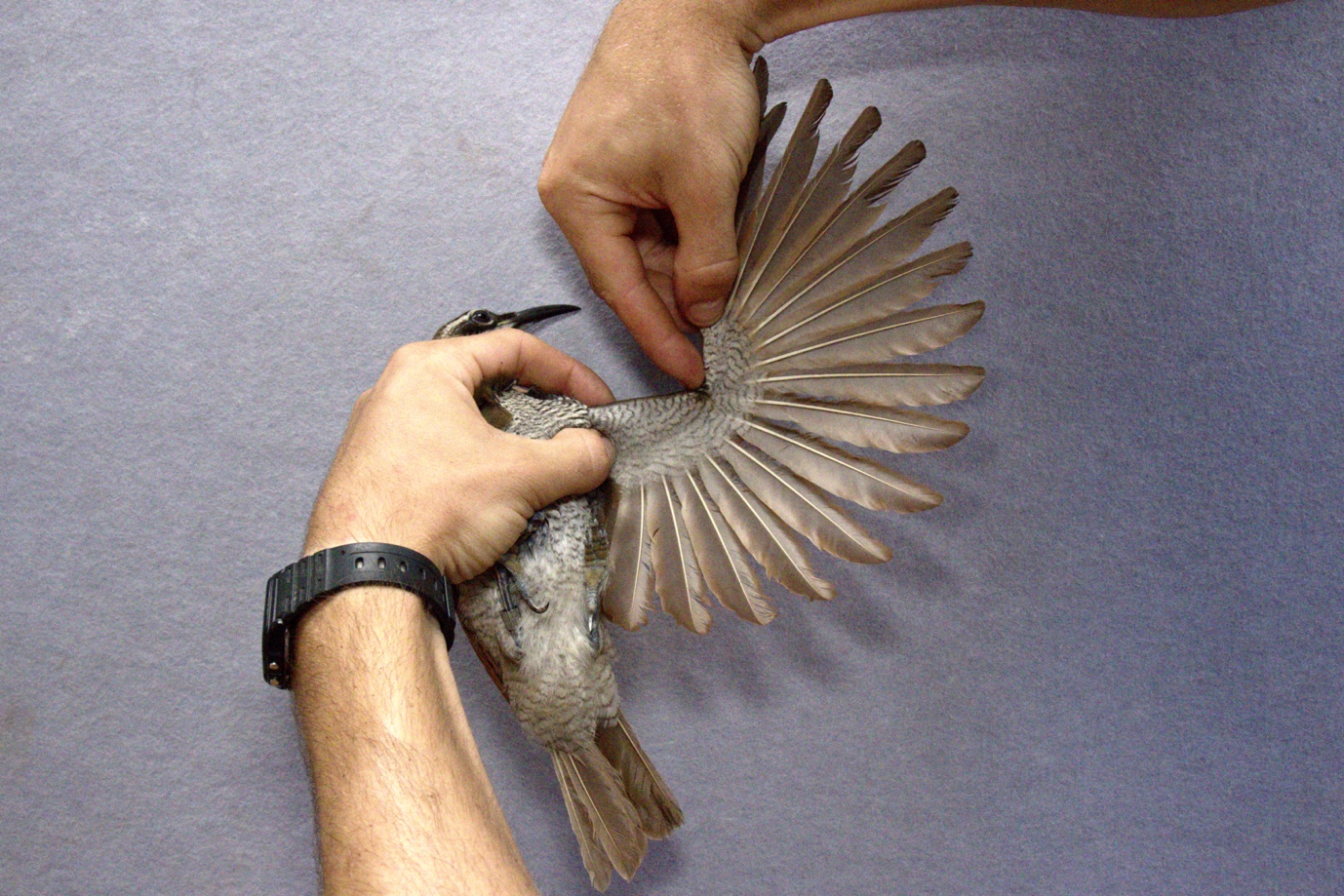


**Figure S4.** Unedited photograph of the adult female Magnificent Riflebird that was measured with wing hyper-extended.


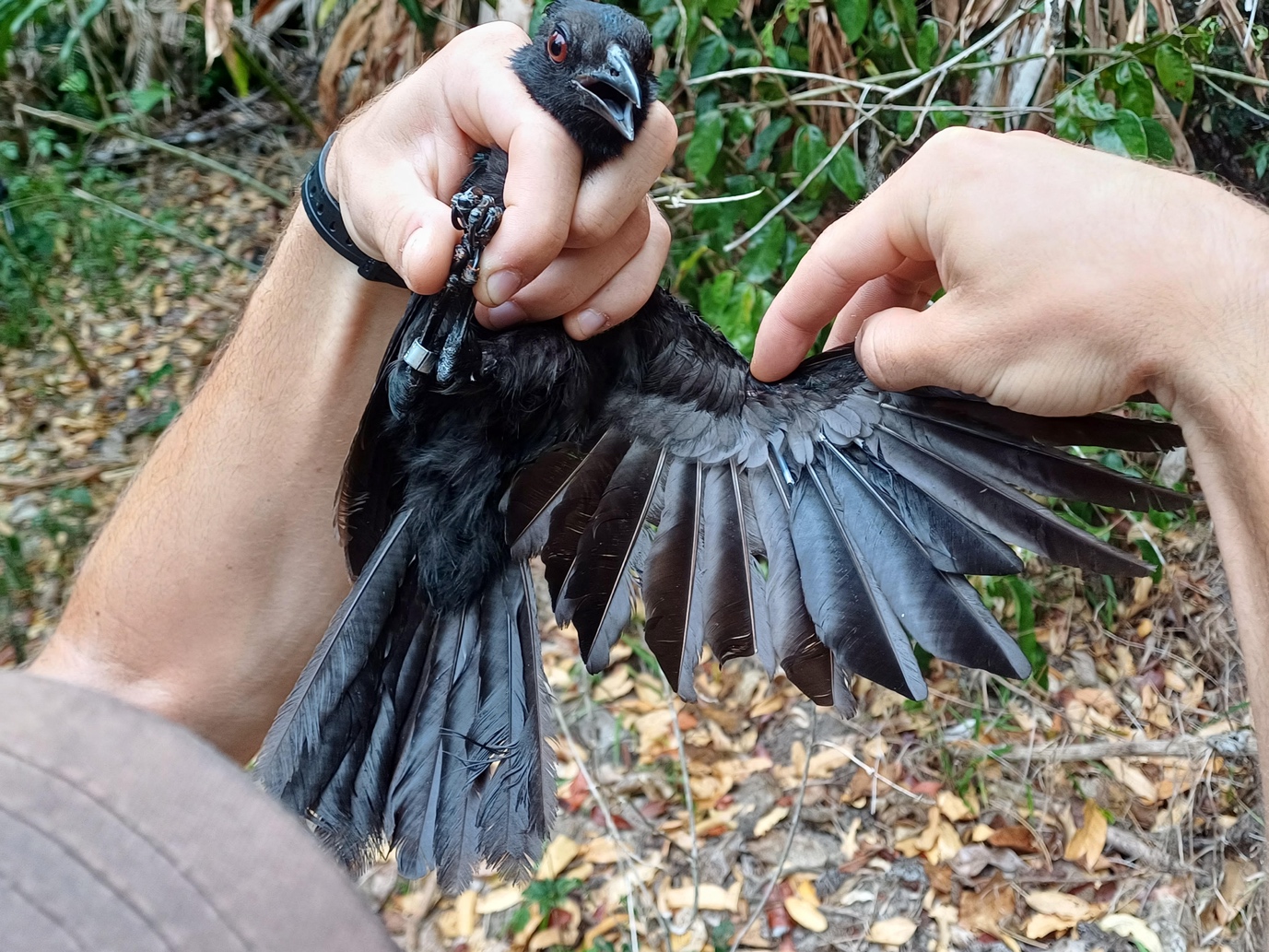


**Figure S5.** Unedited photograph of an unsexed Trumpet Manucode with the wing maximally extended. Note that the alula is somewhat raised and joint positions are not properly indicated, rendering this image unusable for range of motion measurements.

| **Scientific** | **Vernacular** | **Sex** | **Replicate** | **Angle** | **Mean angle** |
| --- | --- | --- | --- | --- | --- |
| *Ptiloris magnificus* | Magnificent Riflbird | Unsexed | 1 | 240.123 | 240.647 |
|  |  |  | 2 | 241.253 |  |
|  |  |  | 3 | 240.566 |  |
| *Ptiloris magnificus* | Magnificent Riflbird | Female | 1 | 239.689 | 236.583 |
|  |  |  | 2 | 234.867 |  |
|  |  |  | 3 | 235.192 |  |
| *Ptiloris victoriae* | Victoria's Riflbird | Unsexed | 1 | 236.491 | 237.067 |
|  |  |  | 2 | 237.375 |  |
|  |  |  | 3 | 237.335 |  |

**Table S1.** Raw measurements of wrist extension taken from the photographs shown in figures S2-4.
